# Combining Diffusion Tensor Imaging and Gray Matter Volumetry to Investigate Motor Functioning in Chronic Stroke

**DOI:** 10.1371/journal.pone.0125038

**Published:** 2015-05-12

**Authors:** Ming Yang, Ya-ru Yang, Hui-jun Li, Xue-song Lu, Yong-mei Shi, Bin Liu, Hua-jun Chen, Gao-jun Teng, Rong Chen, Edward H. Herskovits

**Affiliations:** 1 Department of Radiology, Zhong-Da Hospital, Southeast University, Nanjing 210009, China; 2 Department of Rehabilitation, Zhong-Da Hospital, Southeast University, Nanjing 210009, China; 3 Department of Neurology, Zhong-Da Hospital of Southeast University, Nanjing, 210009, China; 4 Department of Radiology, School of Medicine, University of Maryland, Baltimore, Maryland, 21201, United States of America; University of Surrey, UNITED KINGDOM

## Abstract

Motor impairment after stroke is related to the integrity of the corticospinal tract (CST). However, considerable variability in motor impairment remains unexplained. To increase the accuracy in evaluating long-term motor function after ischemic stroke, we tested the hypothesis that combining diffusion tensor imaging (DTI) and gray matter (GM) volumetry can better characterize long-term motor deficit than either method alone in patients with chronic stroke. We recruited 31 patients whose Medical Research Council strength grade was ≤ 3/5 in the extensor muscles of the affected upper extremity in the acute phase. We used the Upper Extremity Fugl-Meyer (UE-FM) assessment to evaluate motor impairment, and as the primary outcome variable. We computed the fractional anisotropy ratio of the entire CST (CST_ratio_) and the volume of interest ratio (VOI_ratio_), between ipsilesional and contralesional hemispheres, to explain long-term motor impairment. The results showed that CST_ratio_, VOI_ratio_ of motor-related brain regions, and VOI_ratio_ in the temporal lobe were correlated with UE-FM. A multiple regression model including CST_ratio_ and VOI_ratio_ of the caudate nucleus explained 40.7% of the variability in UE-FM. The adjusted R2 of the regression model with CST_ratio_ as an independent variable was 29.4%, and that of using VOI_ratio_ of the caudate nucleus as an independent variable was 23.1%. These results suggest that combining DTI and GM volumetry may achieve better explanation of long-term motor deficit in stroke patients, than using either measure individually. This finding may provide guidance in determining optimal neurorehabilitative interventions.

## Introduction

Stroke is the third most frequent cause of death and the most common cause of acquired adult disability in developed countries.[1] Motor impairment is an important contributory factor to a patient’s ability to live independently. It is a fact that patients who survive a stroke undergo some degree of functional recovery, even in the chronic phase.[2] However, the mechanisms of impairment and recovery are not well understood after stroke.[3]

There is growing interest in the role that central nervous system reorganization might play in the recovery process after stroke.[4] Even in the chronic phase, practice-based interventions could improve affected arm function in the chronic state.[5] Therefore, how to manipulate this reorganization to provide clinical benefits for patients is important. Studies in humans suggest that functionally relevant adaptive changes occur in cerebral networks following stroke[4]. An understanding of how these changes influence the recovery process will facilitate the development of novel therapeutic techniques and will allow the delivery of specific therapies to appropriately targeted patients suffering from stroke.[3]

Magnetic resonance imaging (MRI) is playing an increasingly prominent role not only in identifying ischemic lesions, but also in delineating the relationship between lesions and brain anatomical and functional changes.[6–9] Noninvasive MRI biomarkers may provide insight regarding specific neural events underlying stroke recovery. Furthermore, these biomarkers may prove prognostically useful, and may identify patients who may benefit from specific rehabilitative interventions. [10] The use of MRI biomarkers as surrogate endpoints may facilitate the screening of novel therapeutic interventions[11], provide insights into treatment mechanisms, and allow for more patient-specific treatment.[10, 12]

The importance of the corticospinal tract (CST) in motor recovery has been established using various modalities, such as diffusion tensor imaging (DTI),[8] transcranial magnetic stimulation[13] and magnetic resonance spectroscopy.[14] DTI is one of the most widely used MRI methods to investigate white matter integrity in vivo.[15] DTI allows quantitative analysis of fiber pathways and the anatomy of axonal fiber bundles, and it can identify disease-specific alterations of fiber tracts.[8, 16] Fractional anisotropy (FA), which is derived from DTI, is one of the most commonly used metrics to reflect microstructural status of WM; it quantifies the extent to which water diffusion is directionally restricted, and is influenced by a number of factors, including axonal myelination, diameter, density, and orientation coherence.

Quantitative FA measures of the CST have been correlated with motor impairment in both cross-sectional and longitudinal studies.[16–20] Cross-sectional studies focus on one time point, while longitudinal studies involve two time points. Several cross-sectional studies in chronic patients reported that the structural integrity of the CST after stroke is closely linked to the degree of motor impairment.[17–20] A longitudinal study from Puig and his colleagues found rFA values at day 30 correlated with the degree of motor deficit at 2 years and was an independent predictor of long-term motor outcome after acute stroke in their longitudinal study. [16]

However, current FA measures of the CST only partially explain long-term motor impairment, and considerable variance remains unexplained.[8, 21] Several recent MRI studies suggested that morphologic brain alterations after stroke might also contribute to motor impairment.[22–24] Atrophy in seemingly normal regions of the brain (no signal-intensity abnormality) that are distant from the infarct might have accounted for at least part of the observed sustained motor deficit, and are correlated with motor improvement from constraint-induced movement therapy in chronic stroke.[22] Similarly, a longitudinal study showed that changes in gray matter (GM) volumes, especially in specific motor-relevant brain regions occurring distal to the primary subcortical cerebral infarct, were associated with functional recovery after subcortical cerebral infarct.[23] Another longitudinal study found extensively decreased GM volumes in brain regions bilaterally, including secondary motor-related brain regions that were not associated with motor ability. In addition, increased GM volumes were found in cognition-related brain areas, and were associated with recovery of motor function.[24] In Sterr’s study, cortical thickness in the contralesional primary somatosensory cortex increased and motor function improved with the intervention constraint induced therapy.[25] These studies suggest that morphologic alterations of the brain after stroke may contribute to explanation of long-term motor function.

In summary, there is evidence that both FA values of the CST and GM volumes may be useful in stroke patients with motor impairment. However, no study has systematically evaluated the change of both white matter and gray matter including FA values of the CST and GM volumes to determine whether this more systemic assessment could increases explanation accuracy for motor impairment in chronic stroke patients, relative to using the white matter and GM measurements separately. In addition, multiple linear regression model was used in our study to determine the responsible parameter in motor status in patients with chronic stroke. The result might be important to help decide therapy type and dose not so-called standard care in rehabilitation plan.[26]

We hypothesized that combining DTI and GM volumetry better characterizes long-term motor function in patients with chronic stroke, than using either DTI-derived or volumetric measurements separately. We performed four analyses to test this hypothesis: (1) relating demographic data and disease characteristics to the Upper Extremity Fugl-Meyer (UE-FM) scale; (2) relating FA values in the CST to the UE-FM scale; (3) relating GM volumes of automated anatomical labeling (AAL) atlas[27] brain regions to the UE-FM scale; (4) determining the model that most accurately characterizes motor impairment, using all DTI- and volumetry-derived variables that we found to be significantly associated with the UE-FM scale. We expect that combining FA values and GM volumes will achieve better explanation accuracy of long term motor deficit, as measured by the UE-FM scale, than using either set of variables separately, in patients with chronic stroke.

## Materials and Methods

### Subjects

Participants were recruited from 112 consecutive stroke patients. 31 stroke patients were enrolled according to the following inclusion criteria: (1) > 6 months after stroke onset, (2) age: 20 to 80 years, (3) Medical Research Council strength grade ≤ 3/5 in extensor muscles of the affected upper extremity in the acute phase (first 24 hours after symptom onset), (4) all patients received routine rehabilitation therapy, and (5) all patients were right-handed before stroke. Exclusion criteria were as follows: (1) prior or subsequent symptomatic stroke; (2) bihemispheric infarcts, primary intracerebral hemorrhage, or other disorder that impaired motor function of the stroke-affected hand and leg; (3) other concomitant neurological or psychiatric disease; and (4) contraindication to MRI. Demographic and clinical findings for 31 patients in detail can be seen in Table 1. All patients in this study were treated in accordance with standard procedures of Zhongda hospital, Southeast University and all the patients required inpatient rehabilitation from 1 month to 6 month according to different clinical symptoms. This study was approved by the Ethics Committee of Zhongda hospital, Southeast University and a signed informed consent form was obtained from every subject prior to the experiment.

**Table 1:** Demographic and clinical findings for 31 patients.

Patient No.	Sex	Age	Education(years)	Lesion volume(mm3)	MRC	Lesion-side	UM-FM score	Time after stroke (months)	Lesion location
01	M	67	9	551.2	0	R	25	9.7	PLIC
02	F	64	8	859.3	0	L	13	6.5	CR, tempral lobe
03	M	63	6	1914.0	3	R	4	23.3	PLIC, parietal lobe
04	M	37	17	1429.6	0	R	12	6.9	PLIC, BG
05	M	65	9	1339.0	0	R	15	54.4	CR, BG
06	M	60	9	5133.6	0	R	21	42.2	CR, BG
07	F	69	8	1280.8	3	L	6	12.6	CR, BG
08	M	62	6	392.0	3	R	31	15	BS
09	M	62	6	1592.6	0	L	5	26.2	BG, CR
10	F	73	0	845.0	1	R	8	40.4	BG, CR
11	M	59	15	11777.9	0	L	15	17.3	CR, BG, temporal lobe
12	F	50	1	830.7	3	R	10	6.3	CR, BG
13	M	60	8	575.1	3	R	26	58	CR, BG
14	F	65	0	15763.3	3	R	6	7.3	CR, BG
15	F	57	12	1287.5	2	L	38	40.2	CR, BG
16	M	54	9	144.0	3	L	43	11.9	TH, BS
17	M	68	3	84.9	3	R	44	14.4	BS
18	M	39	12	735.3	2	L	43	6.1	BS
19	F	78	15	1592.6	3	L	60	11.5	PLIC, BG
20	M	73	17	1098.6	3	L	64	107.4	BG
21	F	29	15	4701.6	1	R	61	14.1	BG, CR
22	F	62	8	123.0	2-	R	64	7.4	BS
23	M	57	8	199.3	3	R	61	59.3	BS
24	F	80	19	620.8	3	R	63	11.9	BG, CR
25	M	56	12	832.6	3	R	62	8.2	CR
26	F	58	7	1181.6	3	R	62	7.6	CR, BG
27	M	61	0	96.3	3	R	66	12.7	TH
28	F	60	6	151.6	2	R	15	54.2	BS
29	M	70	14	775.3	3	R	47	7.9	BS
30	M	47	14	11921.9	3	R	17	10.4	CR, BG, frontal lobe
31	M	66	9	10230.1	3	R	9	7.2	CR, BG, tempral lobe

Note: M, male; F, female; L, left; R, right; UE-FM, Upper Extremity Fugl-Meyer assessment; MRC, Medical Research Council; IC, internal capsule; PLIC, posterior limb of IC; BG, basal ganglia; CR, corona radiata; TH, thalamus; CS, centrum semiovale

### Assessment of Motor Function

We assessed motor function with the UE-FM scale;[28] scores ranged from 0 to 66, with higher scores reflecting more complete function recovery. All behavioral assessments were scored on the same day as the MRI examination.

### Image Acquisition

Image data were acquired using a 3.0-T MRI system (Siemens Verio System, Erlangen, Germany). The structure scanning parameters were as follows: T1-weighted sagittal magnetization-prepared rapid gradient echo, repetition time (TR) = 1900 ms, echo time (TE) = 2.48 ms, inversion time = 900 ms, flip angle = 9°, field of view (FOV) = 256×256 mm, voxel dimension = 1×1×1 mm voxels, and slice thickness = 1.0 mm, 176 slices. We also acquired DTI data using a single-shot echo planar imaging (EPI) sequence including 30 non-linear diffusion directions with b = 1000 s/mm^2^ and an additional volume with b = 0 s/mm^2^. The detailed parameters are as follows: number of axial sections, 70; slice thickness = 2.0 mm; gap, none; voxel dimension = 2×2×2 mm voxels; TR = 10,000 ms; TE = 95 ms; FOV = 256×256 mm; matrix = 128×128 and average = 2. To identify the location and size of the lesion, we also acquired fluid attenuated inversion recovery (FLAIR) images: number of axial sections, 20; slice thickness = 5 mm; gap, none; voxel size = 1.3×0.9×5.0 mm; TR = 8500 ms; TE = 94 ms; FOV = 230× 208 mm, flip angle = 150°.

### MRI Analysis

Three-dimensional T1-weighted MRI data were analyzed using Functional Magnetic Resonance Imaging of the Brain’s (FMRIB) Software Library (FSL) (www.fmrib.ox.ac.uk/fsl). The image processing pipeline was as follows. First, the structural MRI images were skull-stripped and segmented into GM, WM, and cerebrospinal fluid. Then we registered a subject’s structural image to the Montreal Neurological Institute (MNI) template, using the FMRIB’s Nonlinear Image Registration Tool (FNIRT)(http://fsl.fmrib.ox.ac.uk/fsl/fslwiki/fnirt). Based on the deformation field generated by the registration algorithm, we parcellated each subject’s T1 brain volume into 90 AAL regions.[27] Visual quality checks are performed by experienced neuroradiologist (M.Y.), and inaccuracies are manually edited and corrected by reprocessing.

To investigate the association between a brain region and motor function, we subsequently calculated the number of voxels in each AAL region and converted it into a volume number (the number of voxels multiple by the voxel size). AAL brain regions were divided into ipsilesional and contralesional regions, to compare stroke hemisphere to non-stroke hemisphere. For a brain structure S such as the hippocampus, VOI_ratio_ of this structure, denoted by VOI_ratio_(S), is defined as the ratio (VOI_ipsi_ / VOI_contra_), where VOI_ipsi_ is the ipsilesional structural volume and VOI_contra_ is the contralesional structural volume. VOI_ratio_ is a relative measurement, which indicates diminished structural integrity in the ipsilesional side after stroke. Although VOI_contra_ could be affected by the infarcts due to secondary degeneration, it does not seem to be severe enough to affect relative measures such as VOI_ratio_, suggesting that these approaches can still detect lesion induced differences in patient groups, which just like the cases used in measurement in FA_ratio_ in chronic stroke patients.[16, 18]

**Fig 1:**
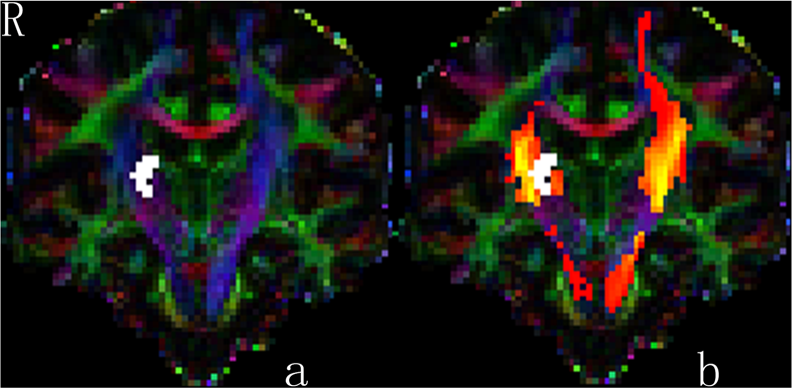
Coronal views of individual color-coded FA map (a) and postprocessed CST map (b) of subject 03 in our study. The patient’s UM-FM score was 25 and time after stroke was 9.7 months. The postprocessed CSTs map (the color of red-yellow in the Fig b) was overlayed with actual CSTs map (the blue color in the Fig a). The infarct (white color) is just inside the right CST and the volume of the ipsilesional CST decreased contrasted with the contralesional one. FA_ratio_ is 0.88. Colors indicate direction of fiber tracts (red, left–right; blue, cranio-caudal; green, anterior–posterior).

DTI processing was performed using FSL, which has been reported in our previous study[29]. The DTI data were corrected for head movement and eddy current distortions with the non-diffusion-weighted image (the b0 image) as a reference image. Diffusion tensor models were fitted independently for each voxel and FA maps were generated. For each subject, the b0 image was registered to the T1 image using a mutual information based algorithm. By concatenating the deformation field from the subject’s b0 image to its T1 image and that from the subject’s T1 image to the MNI space, we obtained a deformation field that normalized the b0 image to the MNI space. We inverted the deformation field from the b0 image to the MNI space; and obtained a deformation field from the MNI space to the b0 image. For each subject, CST was delineated by registering the CST structure defined in the Johns Hopkins University WM template[30] to the b0 image (Fig 1). The Johns Hopkins University WM template is defined in the MNI space. All CST delineation was visually checked by an experienced neuroradiologist (M.Y.), who was blinded to UE-FM scale when checking the images, and was manually corrected if necessary. The fractional anisotropy ratio of the entire CST (CST_ratio_) was computed as the ratio (FA_ipsi_ / FA_contra_), where FA _ipsi_ is the ipsilesional CST FA and FA_contra_ is the contralesional CST FA.

Lesion volume of each patient was determined by an experienced neuroradiologist (M.Y.) who manually outlined the signal abnormality on T1-weighted images slice by slice, using the FLAIR images as an additional guide to confirm the extent of the lesion using MRIcron software (www.mricro.com). The whole lesion volumes were determined by summation across all relevant slices.

### Statistical Analysis

Statistical analyses were performed using SPSS Version 13.0 (Chicago, IL). Sample distributions were examined for normality using Kolmogorov-Smirnov test. For each variable, values that exceed either 75th or 25th percentile by 3 times the interquartile range in each group were considered outliers and were replaced by the sample mean.[31] Pearson correlation analysis was used to determine associations between continuous variables (demographic information, FA_ratio_ and VOL_ratio_) and UE-FM. The association between the UE-FM scale and a categorical variable (sex and side of lesion) was tested with the two-sample t-test. Variables that were significantly associated (*p*-value<0.05)with the UE-FM scores were enrolled independent variables of UE-FM. Besides these variables with *p*-value<0.05, we also added four motor-related supratentorial AAL brain regions (precentral gyrus, supplementary motor area, pallidum and putamen) as enrolled independent variables of UE-FM, based on previous studies of brain regions related to voluntary movements.[32] Then we used a stepwise multiple linear regression model to determine the selected model of long-term motor deficit in patients with chronic stroke. In our regression analysis, the outcome variable was UE-FM measured on the same day as the MRI examination.

## Results

### Demographic and Clinical Findings

Demographic and clinical variables for 31 patients with chronic cerebral infarct are shown in Table 1 and their relationships to UE-FM assessment are summarized in Table 2. UE-FM scores ranged from 4 to 66 (mean, 32.8 ± 22.8). The Kolmogorov-Smirnov test showed that the distribution of UE-FM scores satisfied normality. Infarct volume showed a negative correlation with UE-FM (r = -0.38, *p* = 0.04). No correlation was found with age, chronicity and education. No significant difference was found between UE-FM score and sex or side of lesions.

**Table 2:** Relationships between demographic data, disease characteristics, and UE-FM assessment (n = 31).

Variables		*r*	*p*
Age, y ^*^	60.4±11.2	0.02	0.93
Chronicity, m ^*^	23.2 ± 23.3	0.06	0.75
Education, y ^*^	9.1 ± 5.2	0.34	0.07
Infarction volume, mm^3^ ^*^	2582.8±4080.0	-0.38†	0.04
		*T*	*p*
Sex (male:female)	19:12	-0.20	0.84
Side of lesion (L:R)	9:22	-0.14	0.89

^*^ Data are means ± SD; UE-FM, Upper Extremity Fugl-Meyer assessment;

†correlation is significant at the 0.05 level.

Correlation analyses between CST _ratio_, VOI _ratio_ and UE-FM scales are shown in Table 3. The CST _ratio_ and VOI _ratio_ of caudate, thalamus, temporal pole of superior temporal gyrus and middle temporal gyrus were correlated with UE-FM (r = 0.56, *p* = 0.001; r = 0.51, *p* = 0.004; r = 0.54, *p* = 0.002; r = 0.39, *p* = 0.03; r = 0.46, *p* = 0.01; respectively)

**Table 3:** Relationships between CST _ratio_, VOI _ratio,_ and UE-FM (n = 31).

Brain region	Ratio	*r*	*P*	Brain region	Ratio	*r*	*P*
CST*	0.92±0.06	0.56‡	.001	Cuneus	0.94±0.11	0.13	0.48
Precentral*	0.90±0.15	0.19	0.32	Lingual	1.0±0.10	-0.03	0.88
Frontal_Sup	1.02±0.16	0.22	0.23	Occipital_Sup	1.01±0.11	0.15	0.41
Frontal_Sup_Orb	0.97±0.09	0.22	0.23	Occipital_Mid	0.89±0.38	0.05	0.80
Frontal_Mid	0.99±0.12	0.16	0.41	Occipital_Inf	1.02±0.15	0.19	0.30
Frontal_Mid_Orb	1.06±0.19	-0.039	0.83	Fusiform	0.98±0.12	0.14	0.45
Frontal_Inf_Oper	1.08±0.30	0.17	0.38	Postcentral	0.91±0.11	0.26	0.16
Frontal_Inf_Tri	0.88±0.14	0.08	0.68	Parietal_Sup	0.95±0.19	0.29	0.11
Frontal_Inf_Orb	0.94±0.12	0.13	0.50	Parietal_Inf	0.87±0.58	0.07	0.72
Rolandic_Oper	1.03±0.24	0.31	0.09	SupraMarginal	1.26±0.46	0.06	0.76
Supp_Motor_Area*	1.06±0.15	0.08	0.68	Angular	1.19±0.38	0.10	0.60
Olfactory_L	1.01±0.16	0.32	0.08	Precuneus	0.94±0.10	0.10	0.58
Frontal_Sup_Medial	0.89±0.28	0.007	0.97	Paracentral_Lobule	0.93±0.46	0.06	0.74
Frontal_Med_Orb	1.06±0.22	-0.05	0.81	Caudate*	0.84±0.30	0.51‡	0.004
Rectus	0.95±0.13	-0.02	0.90	Putamen*	1.06±0.33	0.18	0.33
Insula	0.86±0.21	0.29	0.11	Pallidum*	0.97±0.07	0.13	0.49
Cingulum_Ant	0.97±0.09	-0.001	1.0	Thalamus*	0.96±0.20	0.54‡	0.002
Cingulum_Mid	1.02±0.13	-0.04	0.83	Heschl	0.85±0.17	0.31	0.09
Cingulum_Post	0.90±0.57	-0.09	0.62	Temporal_Sup	1.08±0.26	0.21	0.27
Hippocampus	0.95±0.11	0.25	0.17	Temporal_Pole_ Sup*	0.92±0.18	0.39†	0.03
ParaHippocampal	1.10±0.29	0.19	0.31	Temporal_Mid*	0.93±0.06	0.46‡	0.01
Amygdala	0.97±0.13	0.28	0.13	Temporal_Pole_Mid	1.07±0.34	0.32	0.08
Calcarine	0.89±0.27	0.13	0.49	Temporal_Inf	1.03±0.13	0.25	0.17

CST: corticospinal tract; UE-FM, Upper Extremity Fugl-Meyer assessment;

*are enrolled independent variables (related to UE-FM, or motor-related AAL brain regions) in the multiple regression model;

†correlation is significant at the 0.05 level;

‡correlation is significant at the 0.01 level

To assess the explanation power of biomarkers derived only from GM volumetry not including CST, we used both VOI_ratio_ of all structures which were significantly correlated with the UE-FM score, and motor-related AAL brain regions, as independent variables for multiple regression analysis. Stepwise multiple regression analysis selected VOI_ratio_ of thalamus as the most important independent variable; it accounted for 27.1% (adjusted R^2^) of the variability of the motor impairment (Table 4, row 3). The model based only on CST_ratio_ and Caudate_ratio_ accounted for 29.4% and 23.1% (adjusted R^2^) of the variability, respectively (Table 4, row 1 and 2).

**Table 4:** Regression analysis of different independent variables.

Model	Variable	Model Summary		ANOVA		Regression Coefficients		
		R	Adjusted R^2^	F	P Value	B Value	Beta	P Value
1	CST	0.564	0.294	13.500	0.001	201.901	0.564	0.001
2	Caudate	0.507	0.231	10.029	0.004	38.351	0.507	0.004
3	Thalamus	0.544	0.271	12.178	0.002	60.800	0.544	0.002
4	CST +	0.644	0.373	9.908	0.001	140.800	0.393	0.024
	Thalamus					39.670	0.355	0.040
*5	CST +	0.668	0.407	11.289	0.000	162.923	0.455	0.004
	Caudate					28.366	0.375	0.016

UE-FM, Upper Extremity Fugl-Meyer assessment; ANOVA, analysis of variance; CST: corticospinal tract;

1, CST_ratio_;

2, VOI _ratio_ of caudate;

3, VOI _ratio_ of all structures which were significantly correlated with UE-FM and motor-related AAL regions;

4, CST_ratio_ and VOI_ratio_ of thalamus;

5, all variables from lesion volume, DTI and GM volumetry;

*selected model.

When we evaluated the optimal model including all variables, including DTI and GM volumetry, multiple regression analysis yielded a model that included both CST_ratio_ and VOI_ratio_ of caudate; this model accounted for 40.7% of the variability of motor impairment (Table 4, row 5). And also showed that CST_ratio_ is the most important independent variable and VOI _ratio_ of the caudate nucleus is the second.

## Discussion

Our findings may support the hypothesis that combining DTI and GM volumetry to investigate motor status in chronic stroke may result in increased explanation of long-term motor function. A multiple-regression model using biomarkers derived from both DTI (CST_ratio_) and GM volumetry (VOI_ratio_ of the caudate nucleus) explains 40.7% of the variability in the UE-FM score, and CST_ratio_ is the most important independent variable and VOI_ratio_ of the caudate nucleus is the second. The adjusted R^2^ of the regression model with CST_ratio_ as an independent variable is 29.4%, and that of using VOI _ratio_ of the caudate nucleus as an independent variable is 23.1%. Such information can be useful to both clinicians and researchers, to help them focus not only on the ischemic lesion itself, but also on other brain regions that are distant from the infarct but are functionally relevant. Thus, additional targets for therapeutic intervention can be identified.

We find a highly significant relationship between FA_ratio_ of the CST and motor function (r = 0.56, *p* = 0.001). Lower FA_ratio_ of the CST is associated with poorer motor outcomes in patients with hemiparetic stroke.[18] Reduced FA of the ipsilesional CST reflects damaged WM microstructure due to the infarct involving CST, and consequent Wallerian degeneration of the CST remote from the infarct.[5]

Several previous studies associated the pattern of CST damage with motor impairment using DTI. Lindenberg and his colleagues found a linear correlation between posterior limb of the internal capsule FA asymmetry and motor impairment, explaining approximately 50% of the variance of this correlation.[8] Another study based on tract-infarct overlap volume showed that CST damage accounted for approximately 30% of the variability in chronic-stage motor impairment[21]. A study measuring the CST using DTI found associations between FA metrics and motor function; this model explained approximately 27% (using ratio of CST) and 32.3% (using asymmetry of CST) of the variance of motor function when calculated from the template’s CST, and it explained approximately 36.9% (using ratio of CST) and 36.8% (using asymmetry of CST) of the variance of motor ability when calculated from the patient’s CST.[18] We find that the FA ratio of CST accounts for 29.4% of the variability in motor status, which is relatively lower than previous reports. We believe that this discrepancy may have resulted from the different subject sample, sample character (for example, different patients enrolled have difference in handedness, infarct sides, time post-stroke and motor function et al.) and methodology (for example, different post-processing methods, scale of motor impairment and selected calculation index et al.).

Moreover, secondary changes have occurred in our sample of chronic stroke patients since the original infarct, and the explanation ability of CST is not as strong as that in early evaluation of CST.[21–22, 33] However, our finding still highlights the importance of CST integrity in motor ability, in accordance with previous studies.[8–9, 16, 21]

An interesting finding in our study is that volume ratio of caudate nucleus is an important independent variable of motor impairment in stroke patients. The caudate nucleus together with putamen, pallidum and thalamus are part of a complex brain network involving motor behavior.[34] Moreover, the caudate nucleus is an important component of the basal ganglia (BG), which play a major role in voluntary movement, and learning and selection of the most appropriate motor or behavioral programs.[34] The BG receives primary input from the cerebral cortex, and sends output to the brain stem and, via the thalamus, back to the pre-frontal, premotor, and motor cortex.[35] Our results find a correlation between VOI_ratio_ of these motor-related regions (caudate and thalamus) and motor function, which support their role in motor-related functional recovery.

The lower ratio of caudate nucleus volume reflects less functional recovery. Comparison between the stroke patients in our study and 31 matched normal controls shows that the reduced ratio of caudate nucleus volume is caused by atrophy of ipsilesional caudate nucleus, not increased volume of the contralesional caudate (S1 Table). We find significant decreased volume in the ipsilesional caudate nucleus (t = -3.962, *p*<0.001), but no difference in the contralesional caudate nucleus (t = -0.258, *p* = 0.859).

The caudate plays an important role in three of these loops: the oculomotor, dorsolateral, and ventral/orbital circuits.[36] In a related way then, the caudate nucleus has been implicated with voluntary movement, learning, memory and social behavior and plays a critical role in supporting the planning and execution of strategies and behavior required for achieving complex goal[37]. Altered caudate volume has been reported in Alzheimer's disease,[38] attention-deficit / hyperactivity disorder,[39–40] Parkinson’s disease[41] and age-related studies.[42] These findings highlight the role of the caudate nucleus in motor and cognitive function. However, the relationship between the volume of the caudate nucleus and motor deficit has not been reported previously, although the caudate nucleus is part of the extrapyramidal motor system and plays an important role in locomotor control.[43] Taken together, our results may indicate the relationship between the ratio of caudate nucleus volume and motor function, and suggest its possible role in motor status.

In our study, the thalamus is the strongest independent variable when we use only the GM volumetry ratio (including VOI_ratio_, which correlated significantly with UE-FM score, and motor-related AAL brain regions) as independent variables for stepwise multiple linear regression model; it accounts for 27.1% of the variability of the motor status. However, when we include all variables (lesion volume, CST_ratio_, all VOI_ratio_ that are correlated significantly with UE-FM score, and motor-related AAL brain regions) as independent variables for stepwise multiple linear regression model, CST_ratio_ and VOI_ratio_ of caudate are the selected model; and it accounts for 40.7% variabilty of motor impairment. One possible reason may be that there is a significant correlation between VOI_ratio_ of thalamus and CST_ratio_ (r = 0.48, *p* = 0.006), as well as between VOI_ratio_ of thalamus and caudate (r = 0.69, *p* <0.01) whereas no correlation exists between VOI_ratio_ of caudate and CST_ratio_ (r = 0.29, *p* = 0.11). These findings suggest that the thalamus shows similar correlation, but is weaker than CST, in explaining motor function, thereby accounting for the thalamus not appearing in the model selected by stepwise multiple regression.

In our stepwise multiple linear regression model, we found CST_ratio_ was the most important predictor and VOI_ratio_ of caudate was the second. CSTratio together with VOIratio of caudate could account for 40.7% variabilty of motor impairment. CST is the important part of pyramidal tracts, which is one of the most important descending tracts in the central nervous system and involved in voluntary movement. [44] Caudate is a part of extrapyramidal system, which is part of the motor system that causes involuntary reflexes and movement, and modulation of movement.[36] The two predictors in our model imply the dominating role of pyramidal tracts and compensatory role of extrapyramidal system in motor status in patients with chronic stroke.

Our study supports the idea that structural damage from stroke may not only directly affect descending motor pathways (i.e. the corticospinal tract), but may also affect seemingly normal regions (no MRI signal-intensity abnormality) of the brain distant from the infarct, which is consistent with previous findings.[22, 45–46] However, altered volumes of subcortical nuclei post-infarct, and their roles in functional-status explanation, are still ambiguous. Our results have opened an avenue in exploring the role of subcortical nuclei in motor rehabilitation, and may further our understanding of lesion-induced motor deficits and target-based therapy.

Our study has some limitations. First, this is a cross-sectional study; a prospective longitudinal study would provide more insight into time-related brain changes, which in turn would be important in understanding the role of the CST and subcortical nuclei in motor status. Second, this study has a moderate sample size; a larger sample would increase statistical power. Third, we can’t detail the fine change after stroke due to inhomogeneous lesions, which is also a difficult point in current stroke study. More complex model, such as LBA (lesion-based analysis) model and Bayesian approach,[47–48] should be used into the future stroke study, which will help us to understand the mechanism of motor recovery after stroke. Finally, not only infarct lesions themselves but also arm immobilization due to hemiplegia after ischemic stroke could cause change of cortical thickness of the sensorimotor cortex and FA value of the CST.[25, 49] Therefore, different motor status in chronic stroke patients will be another inhomogeneous variance in our study. More homogeneous patients with motor ability would be enrolled to reduce the confounding factor in our future study.

## Conclusions

Our results indicate that combining DTI-derived measures and GM volumetry may improve the ability to explain interpatient variability in motor status. Our findings that not only MRI features of the ischemic lesion itself, but also features of functionally related brain regions that are distant from the original infarct, are associated with motor ability, which suggest that therapies aimed at preserving or improving the integrity of remaining tissue are critical for optimal motor recovery. Improved understanding of factors that attenuate the observed atrophy will identify additional targets for therapeutic intervention except original infarct. [11–12, 22]

## Supporting Information

S1 Table Motor-related supratentorial brain regions volumes in stroke patients and normal controls. (DOC)

S1 Text Demographic information in stroke patients and normal controls, as well as enrolled brain regions volumes (DOC)
