# Supplementary material for: Combining Diffusion Tensor Imaging and Gray Matter Volumetry to Investigate Motor Functioning in Chronic Stroke
Source: PLoS One. 2015 May 12;10(5):e0125038. doi: 10.1371/journal.pone.0125038 (PMC4428789; doi:10.1371/journal.pone.0125038)
Supplement: S1 Table — (DOC) [file pone.0125038.s001.doc]

Table S1 Motor-related supratentorial brain regions volumes

| Brain regions | Stroke patient | Normal control | t | *p* |
| --- | --- | --- | --- | --- |
| Precentral_ipsi(%) | 0. 519±0.082 | 0. 573±0.059 | -2.951 | 0.023* |
| Precentral_contra(%) | 0. 578±0. 058 | 0. 619±0. 053 | -2.903 | 0.018* |
| Supp_Motor_Area_ ipsi(%) | 0. 393±0.056 | 0. 405±0. 048 | -0.876 | 0.538 |
| Supp_Motor_Area_ contra(%) | 0.373±0.056 | 0.390±0.046 | -1.263 | 0.371 |
| Postcentral_ipsi(%) | 0.555±0.071 | 0.622±0.063 | -3.924 | 0.000* |
| Postcentral_contra(%) | 0.609±0.052 | 0.640±0.063 | -2.119 | 0.106 |
| Caudate_ ipsi(%) | 0.181±0.067 | 0.234±0.032 | -3.962 | 0.000* |
| Caudate_ contra(%) | 0.219±0.051 | 0.222±0.045 | -0.258 | 0.859 |
| Putamen_ ipsi(%) | 0.150±0.030 | 0.151±0.032 | -0.145 | 0.885 |
| Putamen_ contra(%) | 0.148±0.034 | 0.147±0.036 | 0.084 | 0.871 |
| Pallidum_ ipsi(%) | 0.123±0.0094 | 0.124±0.0033 | -0.556 | 0.741 |
| Pallidum_ contra(%) | 0.128±0.0071 | 0.126±0.0042 | 1.073 | 0.450 |
| Thalamus_ ipsi(%) | 0.140±0.036 | 0.152±0.020 | -1.584 | 0.275 |
| Thalamus_ contra(%) | 0.147±0.024 | 0.149±0.021 | -0.286 | 0.905 |
| Intracranial volume (mL) | 1330254.58±94577.084 | 1371650.16±128489.708 | -1.445 | 0.308 |

Data are means ± SD; ipsi: ipsilesion; contra: contralesion; FDA multiple comparison testing is used here. * Comparison is significant at the 0.05 level.
