# Supplementary material for: Combining Diffusion Tensor Imaging and Gray Matter Volumetry to Investigate Motor Functioning in Chronic Stroke
Source: PLoS One. 2015 May 12;10(5):e0125038. doi: 10.1371/journal.pone.0125038 (PMC4428789; doi:10.1371/journal.pone.0125038)
Supplement: S1 Text — (DOC) [file pone.0125038.s002.doc]

**Text S1. Demographic information in stroke patients and normal controls, as well as enrolled brain regions volumes**

31 normal subjects were enrolled by a one-to-one match method. No significant differences between the stroke patients and normal controls were observed in age (patients group: mean ± SD, 60.4 ± 11.2 years; control group: mean ± SD, 59.3 ± 11.4 years; *P* = 0.72), education (group 1: mean ± SD, 9.1 ± 5.2 years; group 2: mean ± SD, 10.8 ± 5.0 years; *P* = 0.19) and sex (patients group: M / F, 19/12; control group: M / F, 19/12). All participants had no known neurological or psychiatric diseases, taking psychotropic medications and contraindications to MRI. The same MRI sequences and image processing pipeline for stroke patients were applied to normal controls.

All motor-related supratentorial brain regions volumes (precentral gyrus, premotor cortex, supplementary motor area, caudate nucleus, putamen, pallidum and thalamus) were obtained in normal controls by a one-to-one match method with stroke patients. The ipsilesional hemispheres in chronic patients were contrasted with the same side hemispheres of matched normal controls. Then the other side hemispheres of normal controls were used as contralesional hemispheres and contrasted with contralesional hemispheres of matched stroke patients. To normalize for head size variability, the %GM volume were computed as the percentage of the intracranial volume; that is, %GM = (GM/intracranial volume) ×100%^1^. Motor-related supratentorial brain regions (GM) volumes in both stroke patients and normal controls are shown in Table S1.

**Supporting Information References**

1. Ge Y, Grossman RI, Babb JS, Rabin ML, Mannon LJ, Kolson DL. Age-related total gray matter and white matter changes in normal adult brain. Part i: Volumetric mr imaging analysis*. AJNR Am J Neuroradi*ol. 2002;23:1327-1333.
